# The recovery of touch DNA from RDX-C4 evidences

**DOI:** 10.1007/s00414-020-02407-9

**Published:** 2020-08-26

**Authors:** Noora R. Al-Snan

**Affiliations:** Forensic Science Laboratory, Directorate of Forensic Science, General Directorate of Criminal Investigation and Forensic Science, Ministry of Interior, Manama, Kingdom of Bahrain

**Keywords:** RDX-C4, IED, Touch DNA, Kingdom of Bahrain, Magnetic beads chemistry, Terrorism, GlobalFiler Amplification PCR kit

## Abstract

RDX (Royal Demolition Explosive) is the organic compound with the formula (O2NNCH2)3. It is a white solid material without smell or taste, widely used as an explosive. It is more energetic explosive than TNT, and it was used widely in World War II. The estimated number of RDX-C4 cases in Bahrain ranged between the years 2015–2018 (May) with a total quantity of 370.72 KG in a total number of 38 cases. The effect of explosive RDX-C4 is very massive and can cause many causalities and fatalities among civilians and policemen. These cases consisted of adhesive film with tapes wrapped around RDX-C4 substance (Demolition Charge M112), black batteries, pipes, black bag contained RDX-C4, and in magnetic improvised explosive device (IED). Touch DNA recovery utilized different collection methods, such as nylon swabbing, tape lifting, and direct cutting of certain parts of the samples that were positive of RDX-C4 through DXR Raman Spectrometer. Samples were extracted and purified with magnetic beads chemistry and quantified. Low copy DNA extracts were subjected to a concentration step. DNA extracts were amplified and processed for detection to obtain reliable results using GlobalFiler Amplification PCR kit and run through ABI 3500xL Genetic Analyzer for fragment length determination. We have discovered that RDX-C4 cannot bind to the DNA nor to the solutions used in DNA typing. Thus, it does not cause DNA inhibition or degradation. From this point of view, we were successful in obtaining acceptable and fit results using advanced techniques. This study will be very useful and informative to assist the forensic community in terrorism case applications worldwide as terrorists do not respect geographical boundaries nor ethnicities of the victims, and the use of DNA profiling technology is the most suitable way to identify the terrorists and keep an end to their violence.

**Electronic supplementary material:**

The online version of this article (10.1007/s00414-020-02407-9) contains supplementary material, which is available to authorized users.

## Introduction

RDX (Royal Demolition Explosive) is the organic compound with the formula (O2NNCH2)3. It is a white solid material without smell or taste, widely used as an explosive. Chemically, it is classified as a nitramide and like HMX, an octogen [1]. It is a more energetic explosive than TNT, and it was used widely in World War II [2]. It is often used in mixtures with other explosives and plasticizers or phlegmatizers (desensitizers) [3]. RDX is very stable at room temperature. It burns rather than explodes. It detonates only with a detonator, being unaffected even by small arm fire. Thus, it is considered one of the most energetic military explosives; and an explosion can only be initiated by a shock wave from a detonator [1].

RDX is the main component of Composition-4 (C4), which is a plastic explosive used in military operations. C4 is composed of RDX (91%), dioctyl sebacate (5.3%), polyisobutylene (2.1%), and mineral/motor oil (1.6%) [3], and the ratios of composition depend on the source of manufacturing. As RDX is the main component of C4, C4 has the explosive nature of RDX and has been utilized due to its malleable nature, which allows it to be molded into any desired shape and redirect the direction of the resulting explosion [4]. Terrorists-related crimes have increased over the past 15 years and have become the most common activity around the world besides theft and sexual assault cases [5].

In every year, terrorists have improved their tools and skills which emphasizes on the importance of having similar skill level of the law enforcements to reduce the challenges upon examining these types of cases. One of the terrorists involved activities is to plant remotely controlled explosive devices known as improvised explosive devices or IEDs in different areas to ensure maximum damage to the communities [6]. The effect of explosive RDX-C4 is very massive and can cause many causalities and fatalities among civilians and policemen. It can penetrate through metals and buildings. Terrorists do not respect geographical boundaries nor ethnicities of the victims, and the uses of DNA profiling technology are the most suitable way to identify the terrorists and keep an end to their violence.

The use of DNA in a criminal investigation such as an incident involving a remotely detonated IED may provide a strong association between a terrorist and the materials used to construct or detonate the device [7]. As the suspected person comes into direct contact with the IEDs in terms of manufacturing, assembly, deploying and transportation. Thus, it is more likely that the wanted person(s) will be identified using touch DNA from different components of the IEDs [7]. Previously in year 2019, we have identified the potential recovery of touch DNA from different types of IEDs. We have proposed a methodology known as forensic DNA intelligence to identify the prepositions of hidden DNA from different parts of the IEDs such as the tapes, wires, batteries, phones, devices, as well as different solid parts [8].

**Fig 1:**
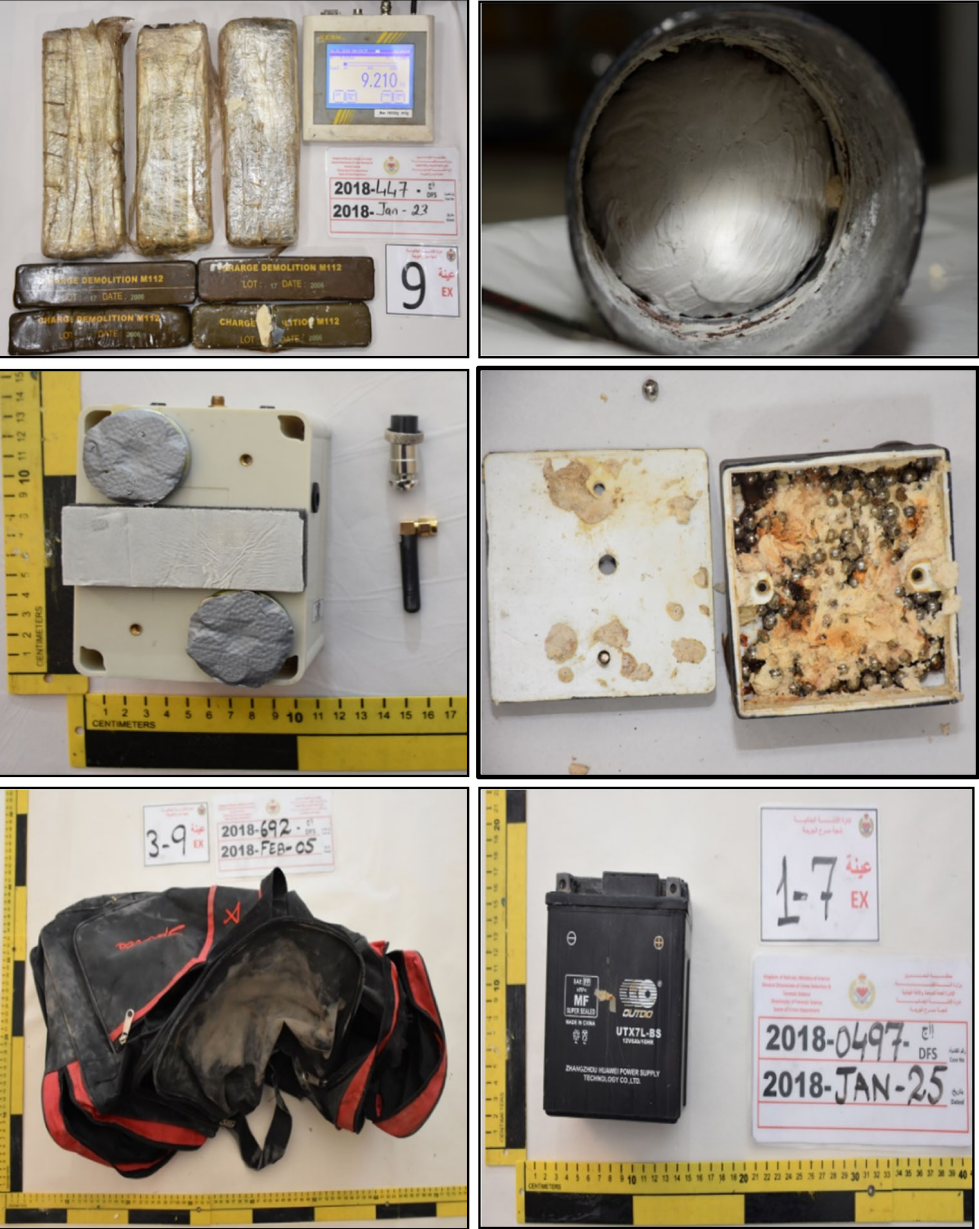
Some of the processed samples of different terrorism cases

Many terrorism cases have shown the presence of RDX-C4 in samples such as real IEDs, bombs, pipes, and some packed in bags or wrapped in adhesive film in warehouses. The estimated number of RDX-C4 cases in Bahrain ranged in between the years 2015–2018 (May) with a total quantity of 370.72 KG and a total number of 38 cases as shown in supplementary material (Fig 1S.).

This dangerously large quantity of RDX-C4 can cause major disaster to the infrastructure and human casualties which we emphasize on the crucial need to continuously re-evaluate standard operating protocols with empirical studies for such type of cases. In this paper, we will display the potential recovery of touch DNA from evidences contaminated with small and large traces of RDX-C4 with no effect of inhibition or degradation of DNA.

## Materials and methods

### Samples collection, extraction, and normalization

Total number of five cases were selected and brought to the Forensic Science Laboratory, General Directorate of Criminal Investigation and Forensic Science, Kingdom of Bahrain during the years 2017 and 2018 (Fig. 1). These samples were seized and found within hidden warehouses and some roads, ready to be deployed. All samples were made safe by the Special Force Team by separating the parts of the IEDs and signing the consent that the samples are safe to send to the Forensic Lab. These cases consisted of adhesive films with tapes wrapped around RDX-C4 blocks, black battery, pipes loaded with RDX-C4, black bag which contained RDX-C4, and finally magnetic IEDs, which were used to target specific vehicles. Samples were previously tested positive for bulk analysis of RDX-C4 using DXR Raman Spectrometer (Thermo Fisher Scientific, Inc., Waltham, MA, USA), as shown in supplementary material Fig 2S.

Different collection methods were applied, such as tape lifts, nylons flocked swabs, and direct cutting of small pieces of sample. Table 1 shows summary of the collection method applied. Tape lifts and single or double nylon swabs (moistened with DNA grade purified water) were used to collect from the samples with RDX-C4 such as from handles and zipper of bag, battery body that was part of IED, pipe’s opening, and from the internal parts of the magnetic (IED). Direct cutting of samples was done to small pieces of tape endings and wire twists inside of the magnetic (IED) [9].

**Table 1:** Summary of the collection method applied to the samples

Collection methods	Samples
Tape lifts	Cloth bag parts inside and outside, battery edges, pipe’s opening, internal parts of magnetic IED
Nylons flocked swabs	Cloth bag parts inside and outside, battery edges, pipe’s opening, internal parts of magnetic IED, parts of adhesive film around Charge Demolition M112
Direct cutting	Tape endings, wire twists inside the magnetic IED

Touch DNA was extracted and purified using the magnetic beads chemistry (i.e., EZ1 Advanced XL, Qiagen, Germany) and AutoMate Express DNA Extraction System , Thermo Fisher Scientific, Inc., Waltham, MA, USA) [10] with increase time of incubation in EZ1 to 60 min at 56 °C, 850 rpm using 475 μl of undiluted G2 buffer and 25 μl PK. All the samples were added to the Investigator Lyse and Spin tubes (Qiagen, Germany) prior to incubation [11].

Quantification was done through Investigator Quantiplex Hyres Kit, Qiagen, Germany or Quantifiler HP DNA Quantification Kit (Thermo fisher Scientific, Inc., Waltham, MA, USA) using a 7500 Real-Time System (Thermo fisher Scientific, Inc., Waltham, MA, USA) following the manufacturers protocols [12]. Most of the samples were subjected to various concentration steps using vacuum dry technique (i.e., using Concentrator Plus – Eppendorf) [13], to obtain a reliable quantity for a successful PCR.

### Amplification and detection

About 1.0 ng of the extracted DNA was amplified using GlobalFiler PCR Amplification Kit (Thermo Fisher Scientific, Inc., Waltham, MA, USA) according to manufacturer’s recommendation in 29 cycles via MicroAmp Optical 96-Well Reaction Plate (Thermo Fisher Scientific Company, Carlsbad, USA) along with the provided positive control and low TE buffer as a negative control in a 96-Veriti thermal cycler (Thermo Fisher Scientific Company, Carlsbad, USA).

A total of 24 loci were amplified, including 21 autosomal STR loci (D8S1179, D21S11, D7S820, CSF1PO, D3S1358, TH01, D13S317, D16S539, D2S1338, D19S433, VWA, TPOX, D18S51, D5S818, FGA, D12S391, D1S1656, D2S441, D10S1248, D22S1045, and SE33) and three gender determination loci (Amelogenin, Yindel, and DYS391).

The PCR products (1 μl) were separated by capillary electrophoresis in an ABI 3500xl Genetic Analyzer (Thermo Fisher Scientific Company, Carlsbad, USA) with reference to LIZ600 size standard v2 (Thermo Fisher Scientific, Inc., Waltham, MA, USA) in a total of 9 μl of LIZ600 standard and Hi-Di formamide (Thermo Fisher Scientific, Inc., Waltham, MA, USA) master mix. GeneMapper ID-X Software v1.4 (Thermo Fisher Scientific, Inc., Waltham, MA, USA) was used for genotype assignment.

DNA typing and assignment of nomenclature were based on the ISFG recommendations. Experiments were performed in the Biology and DNA Forensic Laboratory, Ministry of Interior, Kingdom of Bahrain, which is accredited with CTS testing in regular basis using different quality standard protocols.

### Data analysis

Data was captured by 3500 Series Data Collection v3.1. The raw data was then analyzed using GeneMapper ID-X v1.4. RFU values were obtained through in-house validation of the GlobalFiler PCR Amplification Kit with an average of 85 for each dye. Single source samples were checked into the Bahrain DNA database containing ~ 90,000 DNA STR profiles.

DNA mixtures of 2–3 contributors are supported with likelihood values using LRmix Studio available online [14].

## Results and discussion

Full profiles were generated from the different exhibits containing RDX-C4 as shown in supplementary material (Fig. 3-7). The RFU of the samples were acceptable and fit for interpretation as well as approved by our internal validation for GlobalFiler PCR Amplification Kit (~ 85 for each loci). All the samples generated were of DNA mixtures except for the tape wrapped around the adhesive film of RDX-C4 Charge Demolition M112. The quantity of retrieved DNA was displayed in Table 2.

**Table 2:** Quantity of retrieved DNA from RDX-C4 evidences

Samples	Quantity of DNA (ng/ul)
DNA retrieved from handles of black bag contaminated with RDX-C4	0.75
DNA retrieved from RDX-C4 inside the black pipe	0.001
DNA retrieved from RDX-C4 inside the magnetic IED	0.001
DNA retrieved from tape found on the Demolition Charge M112 (external surface) contaminated with RDX-C4	0.75
DNA retrieved from black battery (external surface) contaminated with RDX-C4	0.75

Many terrorism tactics have recently been developed, from Molotov bottles to handmade local weapons and different types of IEDs. These threats need to have optimized way of identifying through specialized forensic team such as the following [8]:

- Crime scene examiner
- DNA expert
- Fingerprint expert
- Ballistics expert
- Forensic electrical engineer
- Forensic photographer

Thus, each forensic specialist has a significant role in identifying the forensic leads which would assist in the identification of how IEDs and components were manufactured or modified and how they were used. RDX-C4 sample is mostly kept for chemical analysis and most of the labs neglect the crucial need to swabbing or tape lifting the RDX-C4. We have revealed that the RDX-C4 is a sticky solid substance that can retain some of the cells from the shedders or from sweating while assembling the explosive material inside compartments of IEDs or bombs. Also, C4 is very stable and insensitive to most physical shocks and can withstand different physical properties such as welding and molding thru metals and electronics [15]. Thus, collecting DNA from RDX-C4 sample will give a forensic lead to directly identify the suspect(s) who manufactured the IEDs.

We have also discovered that C4 cannot bind to the DNA nor to the solutions used in the protocols. Thus, it does not cause inhibition or degradation to the DNA. From this point of view, we were successful in obtaining acceptable and fit results using the above described methods.

In addition, this study will be particularly useful and informative to assist the forensic community in terrorism case applications worldwide. The findings of this study emphasize the need to continuously re-evaluate standard operating protocols with empirical studies for such type of cases.

## Conclusion

This paper indicates the importance of proper handling of RDX-C4 samples as these samples have high potential of touch DNA stick to the physical nature of the explosive material. We have generated many acceptable and fit STR profiles using the technology mentioned in the paper. It is first published paper to declare the crucial need of focusing upon RDX-C4 to be used in forensic DNA typing application along with Forensic chemistry.

## Electronic supplementary material

ESM 1 (DOCX 11979 kb)
