# Supplementary material for: The recovery of touch DNA from RDX-C4 evidences
Source: Int J Legal Med. 2020 Aug 26;135(2):393–7. doi: 10.1007/s00414-020-02407-9 (PMC7870765; doi:10.1007/s00414-020-02407-9)
Supplement: Supplementary file 1 — (DOCX 11979 kb) [file 414_2020_2407_MOESM1_ESM.docx]

**Fig 1S. Number of RDX-C4 cases in Bahrain from 2015-2018 (May)**


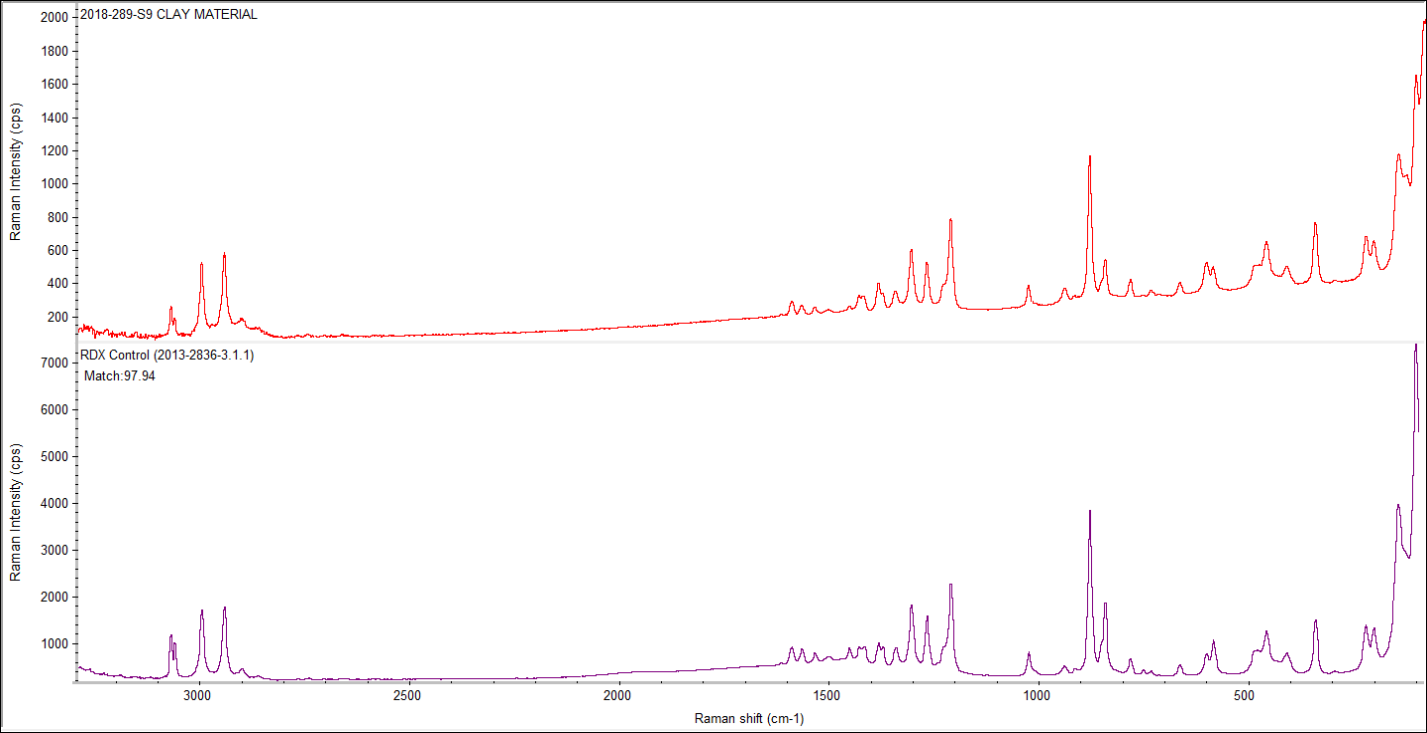


**Fig 2S. Chemical analysis of Evidence showing 97.94 match with RDX control using DXR Raman Spectrometer**


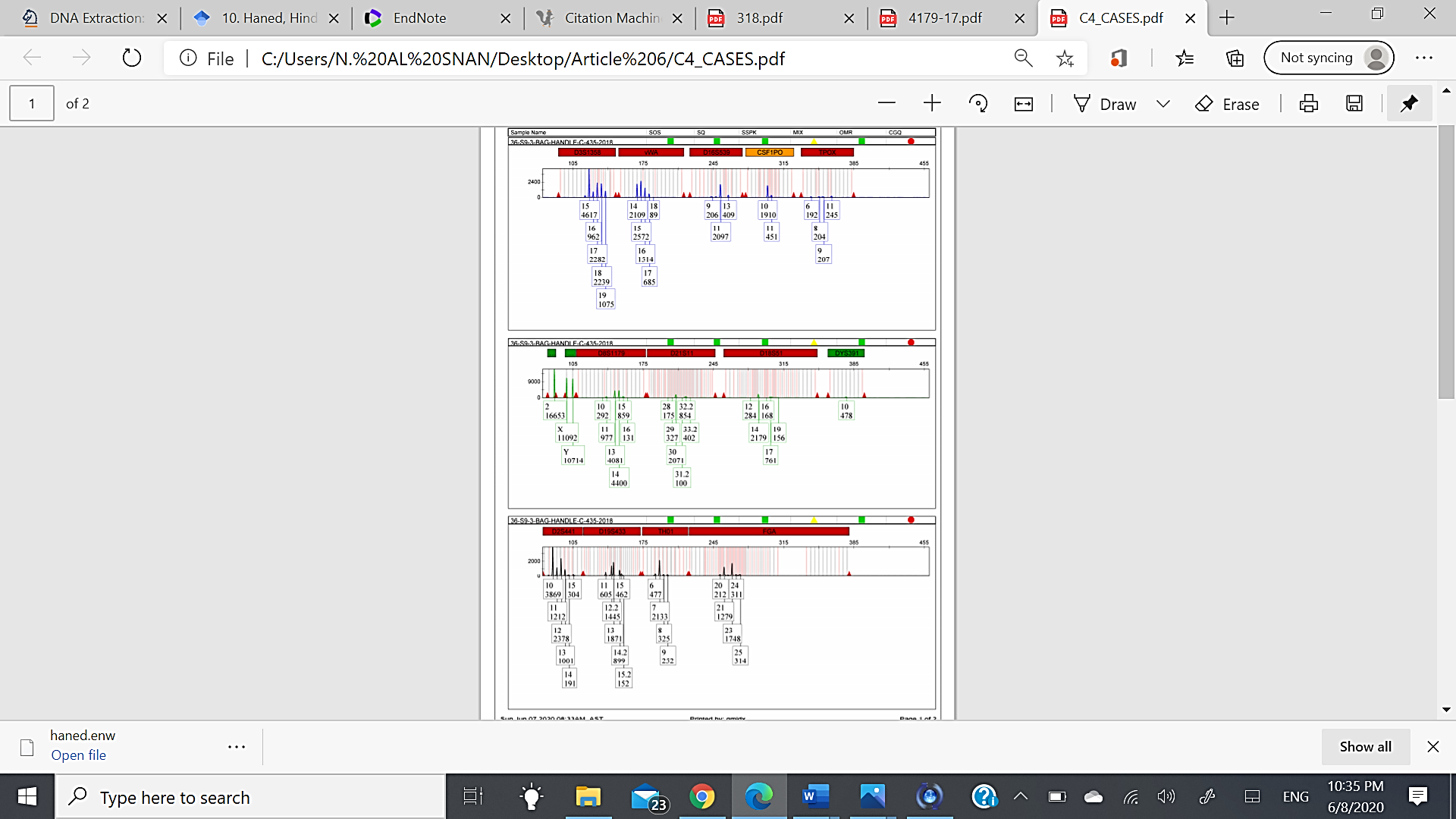

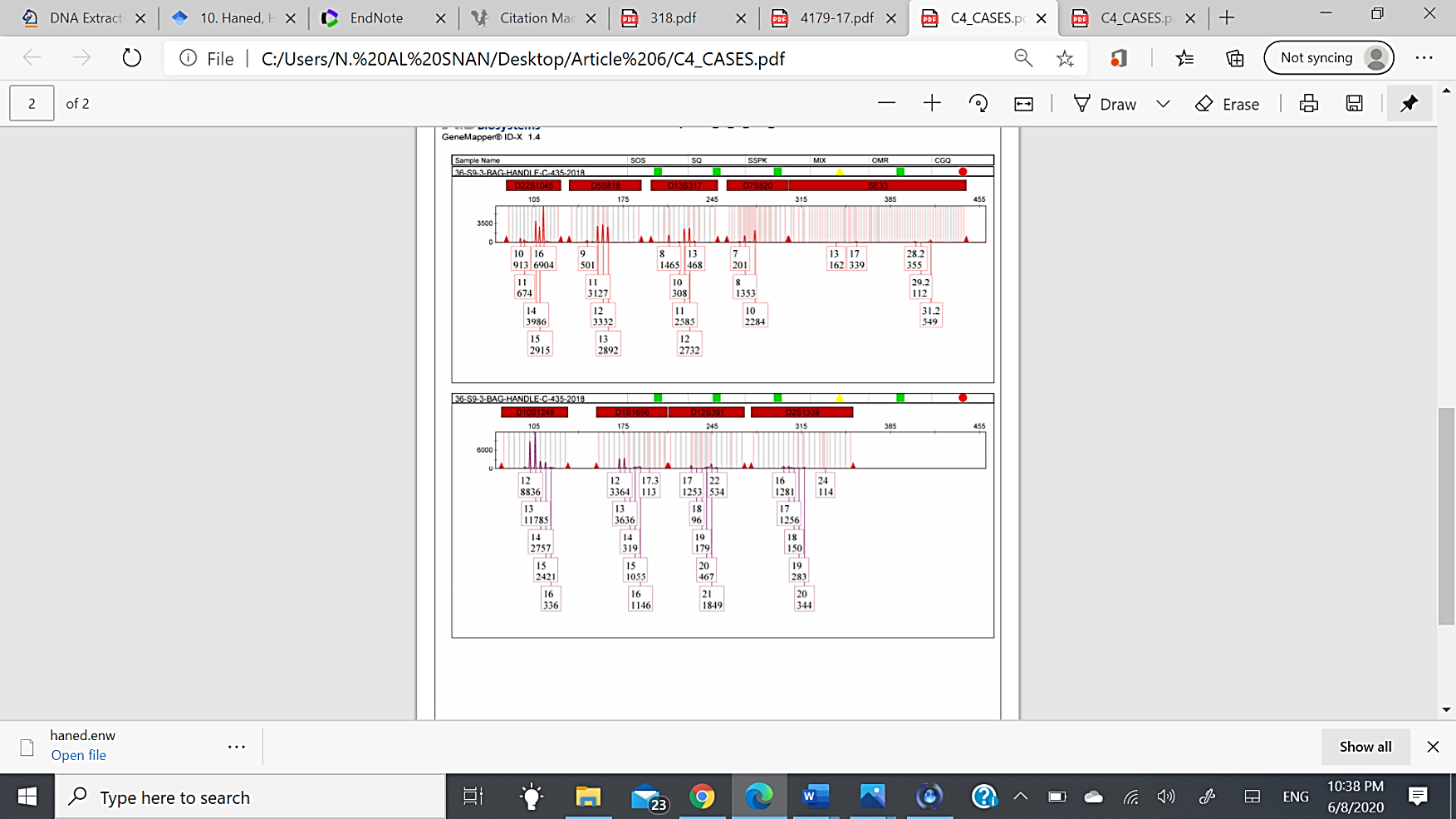


**Fig 3S. The results obtained from handles of black bag contaminated with RDX-C4 showed DNA mixtures (0.75ng/µl)**


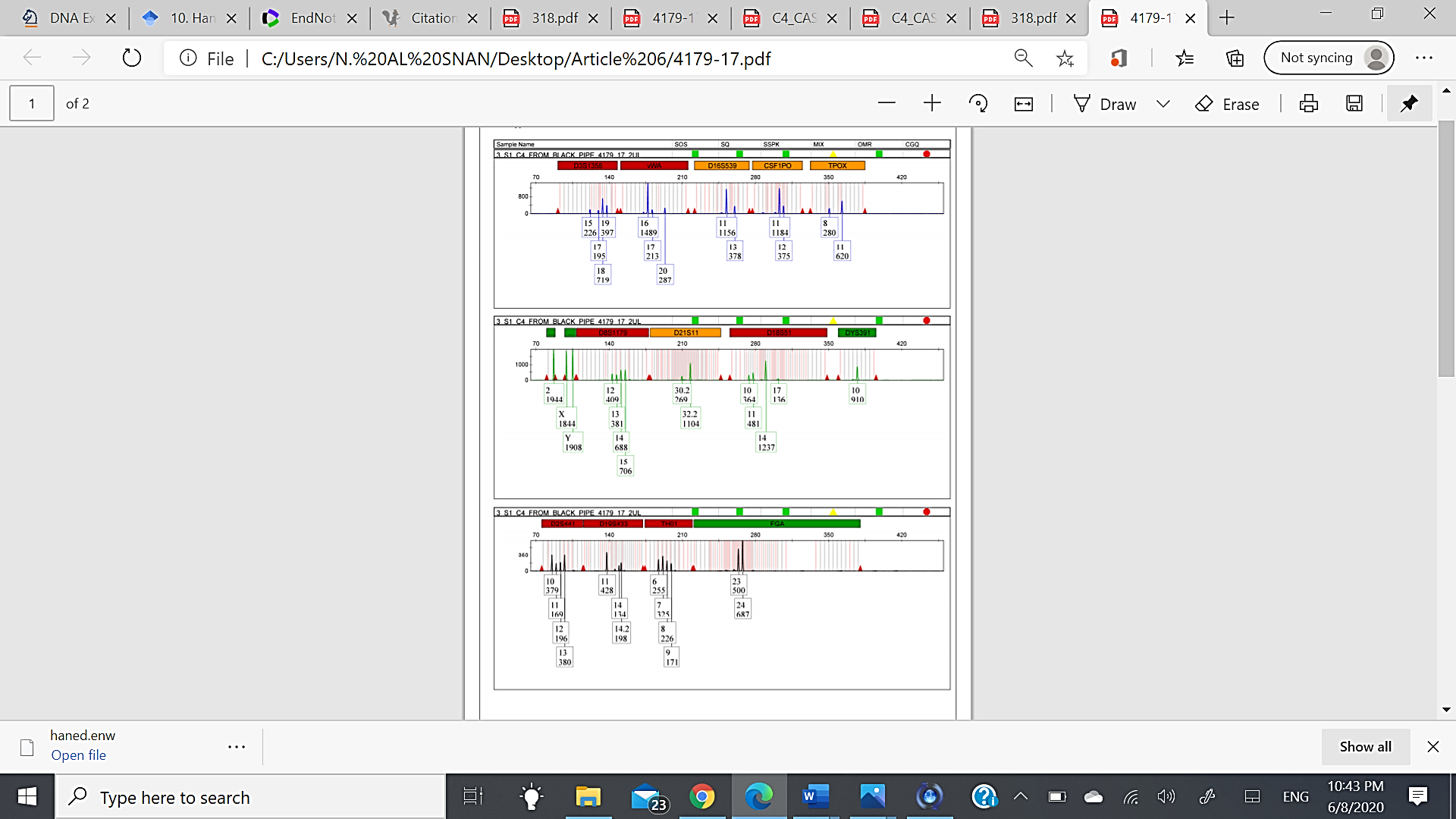

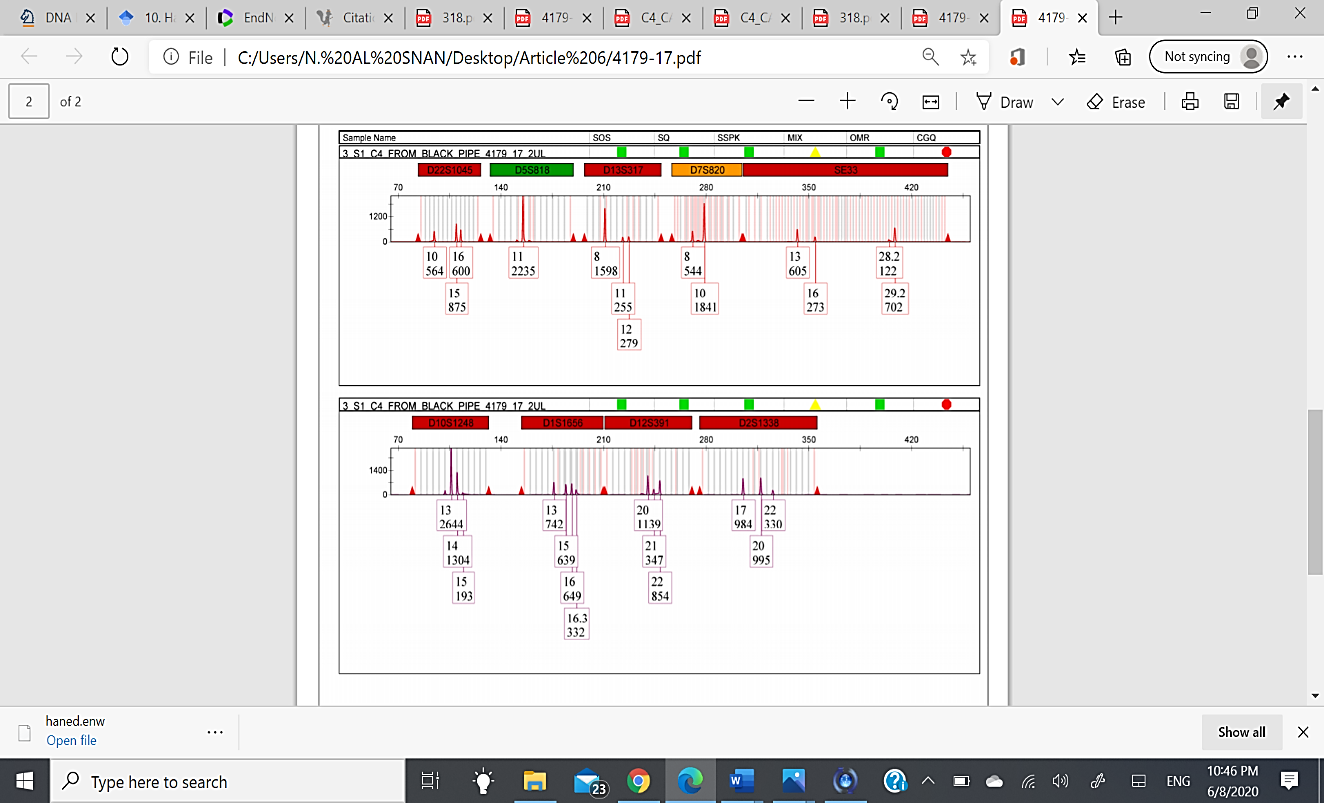


**Fig 4S. The results obtained from RDX-C4 inside the black pipe showed DNA mixtures (0.001ng/µl)**

Figure 4 Figure 4 The results obtained from RDX-C4 inside the black pipe showed DNA mixtures (0.01ng/µl)


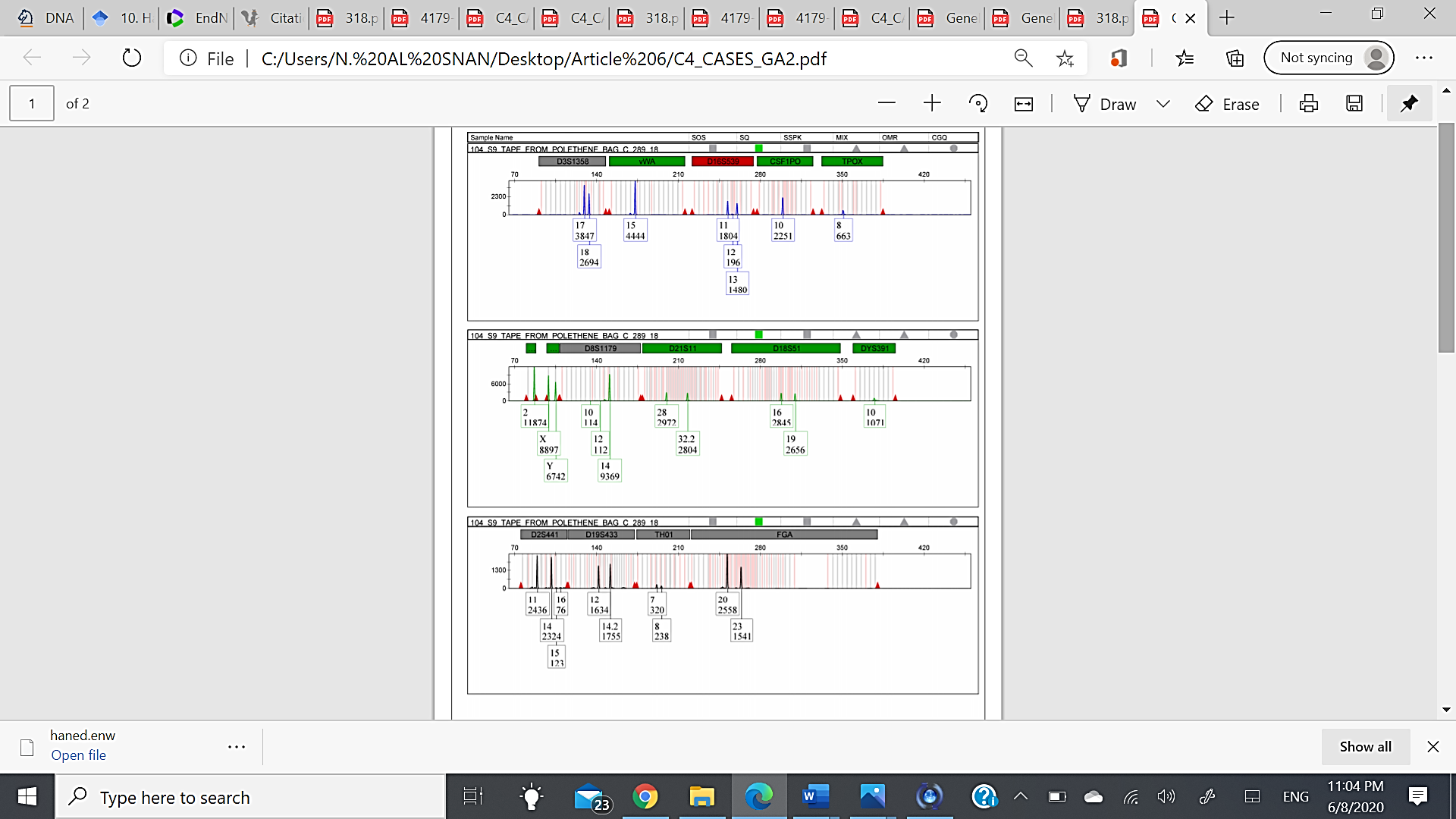

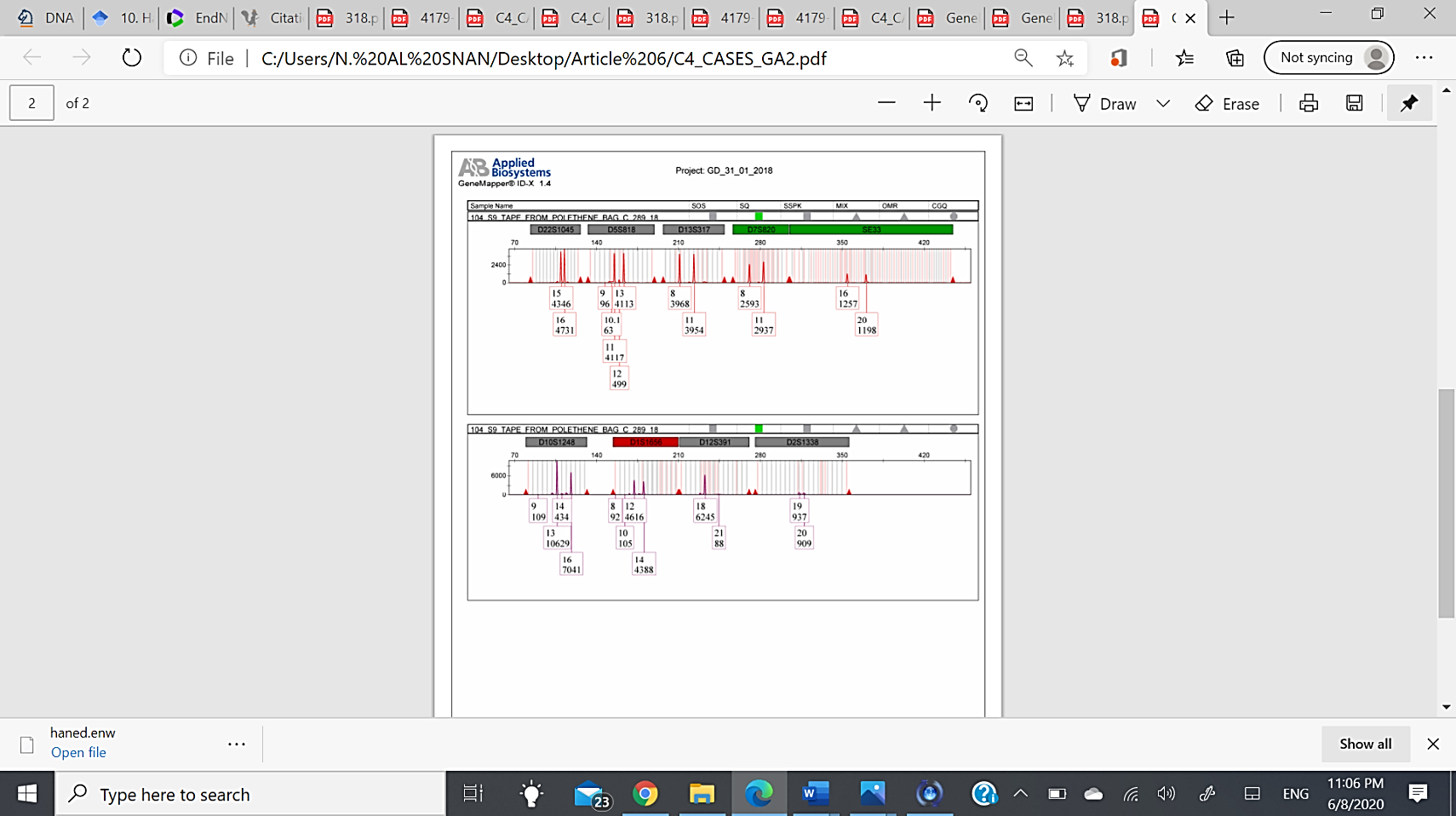


**Fig 6S.** **The results obtained from tape on the Demolition Charge M112 (external surface) contaminated with RDX-C4 showed major contributor of single-source male DNA (0.75ng/µl)**


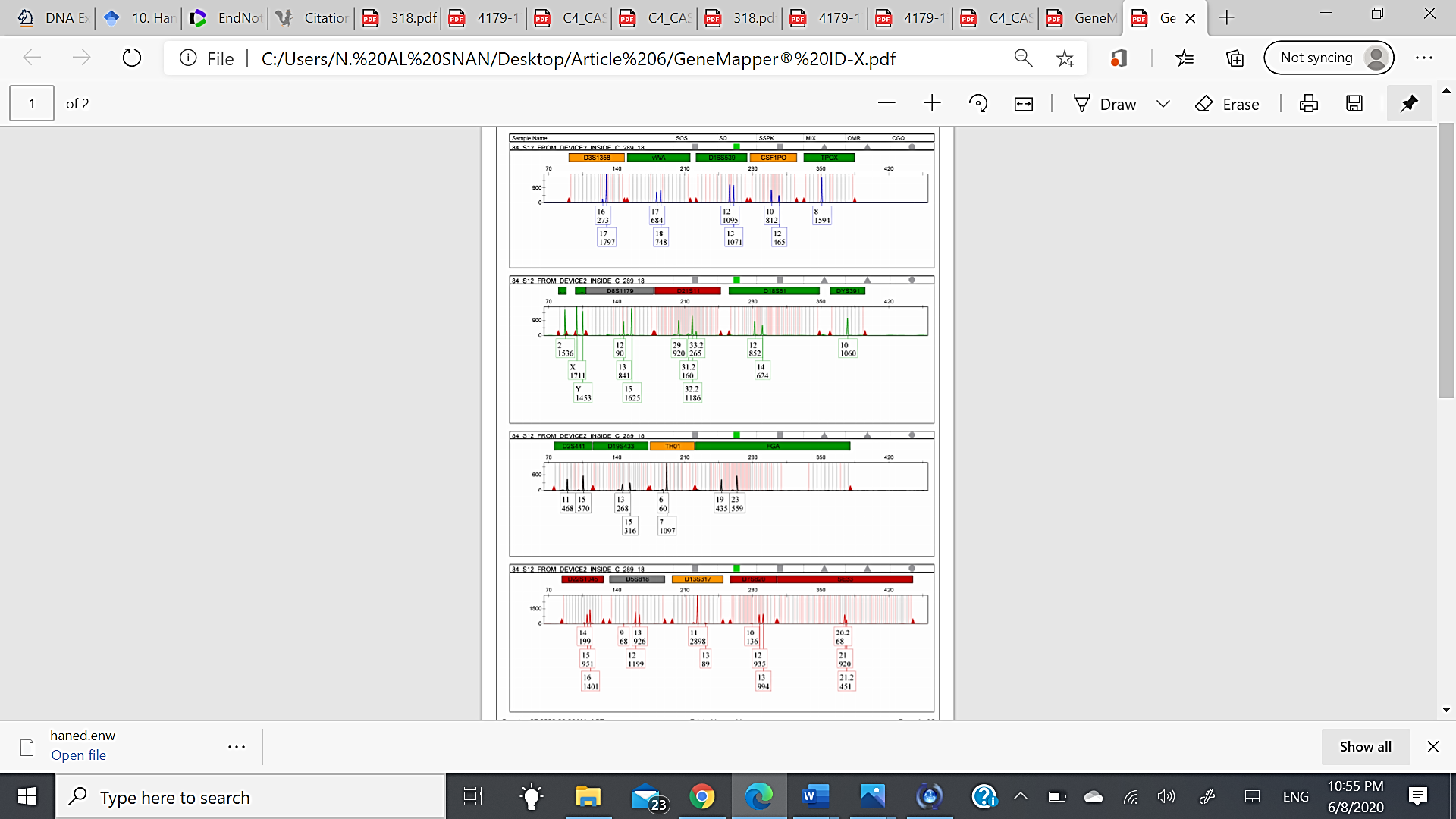

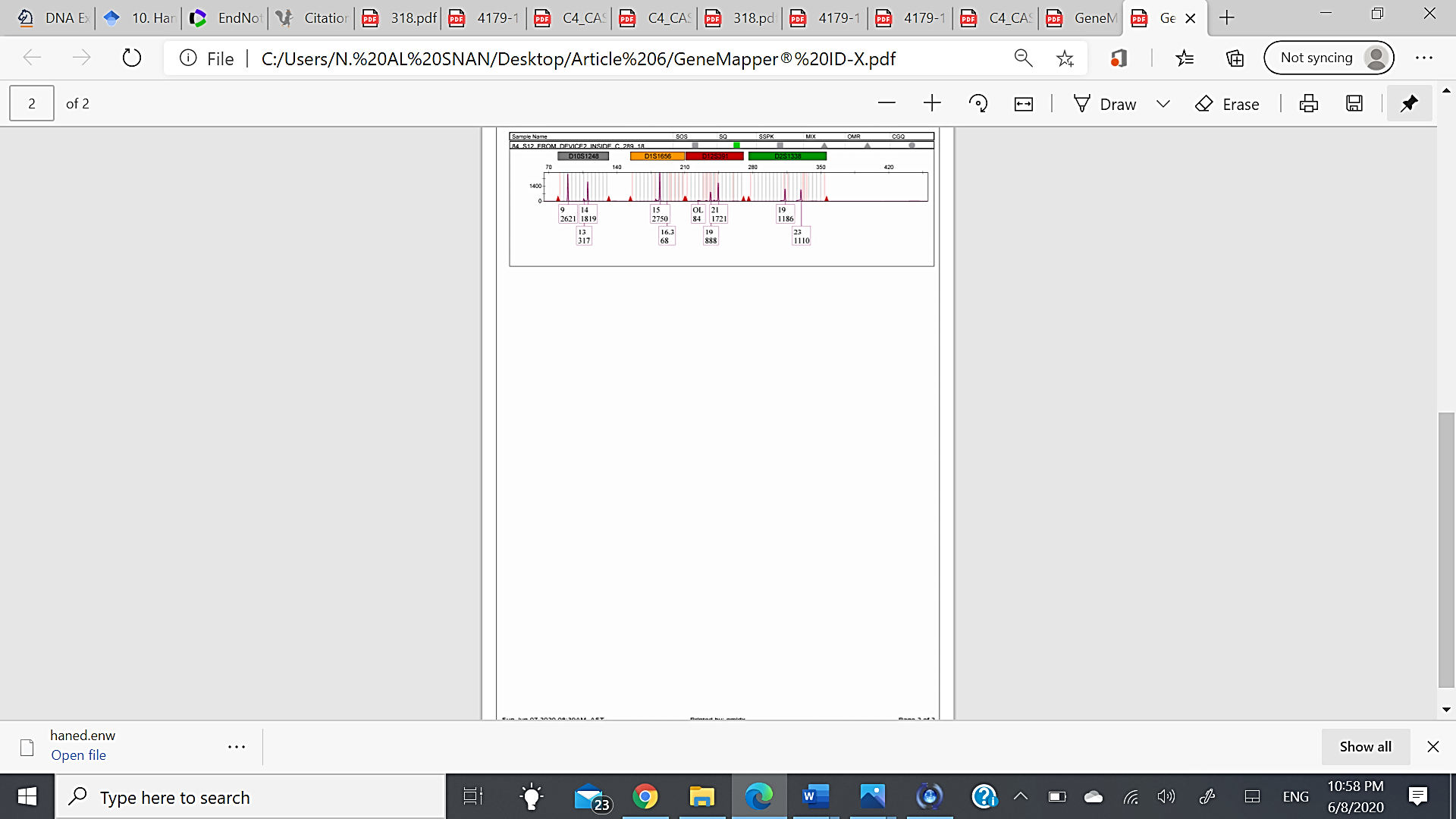


**Fig 5S. The results obtained from RDX-C4 inside the magnetic IED showed DNA mixtures (00.01ng/µl)**


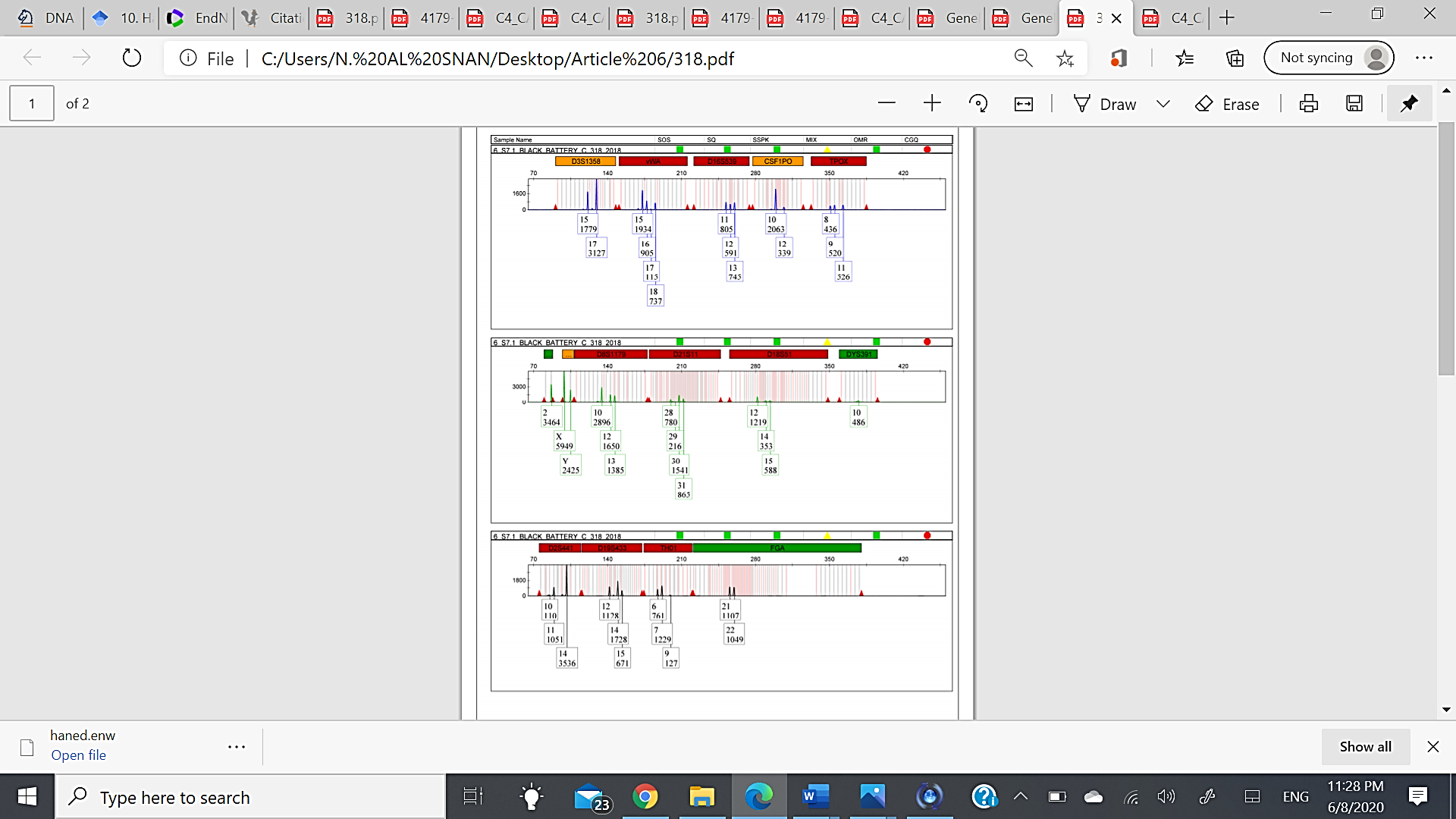

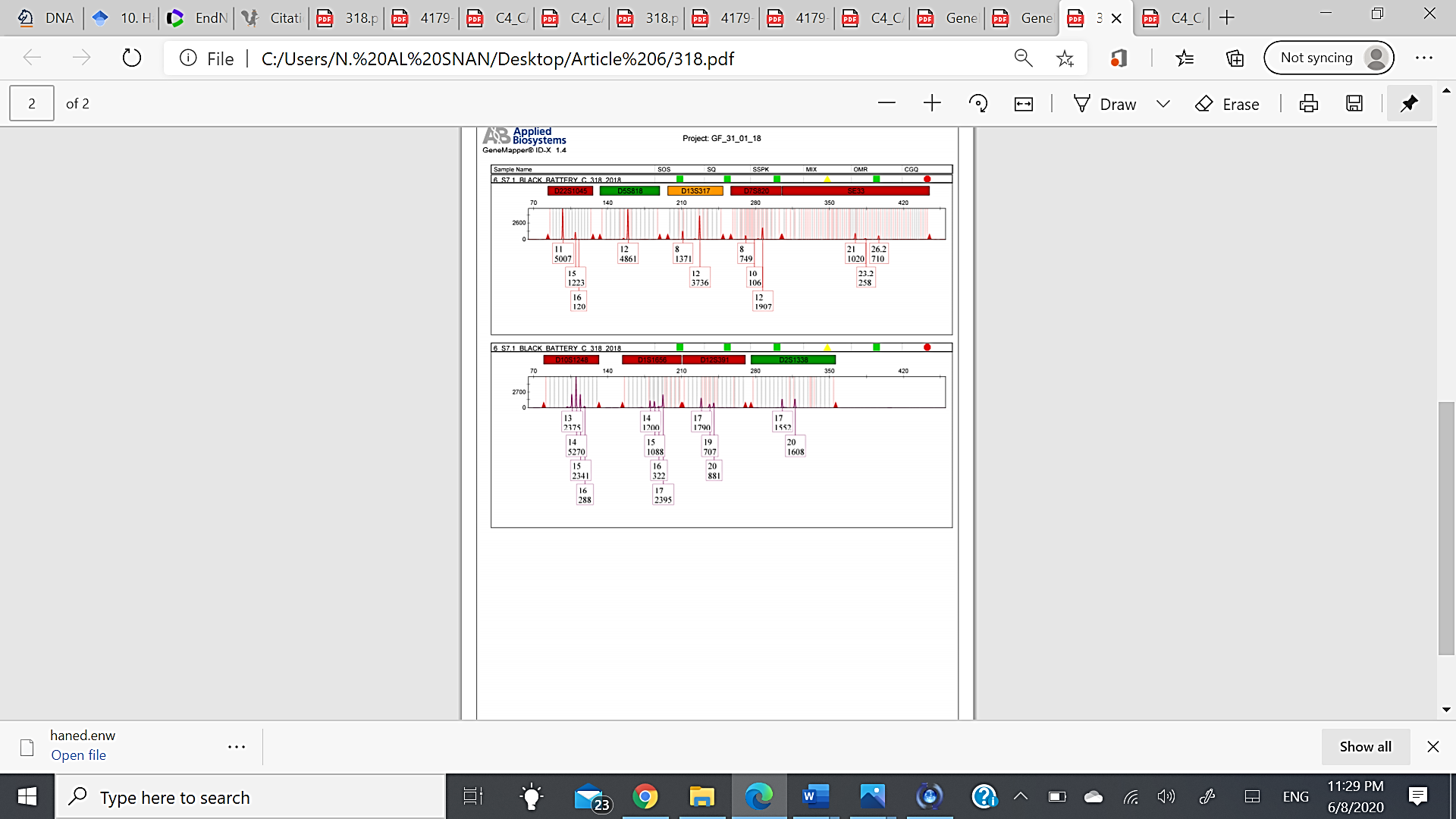


**Fig 7S. The results obtained from black battery (external surface) contaminated with RDX-C4 showed DNA mixtures (0.75ng/µl)**
